# Editorial: PCOS: from infertility to pregnancy

**DOI:** 10.3389/fendo.2023.1220014

**Published:** 2023-05-30

**Authors:** Stefano Palomba, Didier Dewailly

**Affiliations:** ^1^ Sapienza University of Rome, Rome, Italy; ^2^ Université de Lille, Nord-Pas-de-Calais, France

**Keywords:** cardiovascular risk, infertility, PCOS, polycystic ovarian syndrome, pregnancy, reproduction

Polycystic ovary syndrome (PCOS) is a heterogeneous and very frequent endocrinological disease associated with reproductive alterations (1). Women with PCOS have reduced fertility in the presence of ovulatory dysfunction that is a key element in the pathogenesis of PCOS (2). However, patients with PCOS have alterations in several reproductive athways independently of their ovulatory status (3). These include primary and secondary alterations of the oocyte and embryo quality (4), and of endometrial competence (5). Those endometrial alterations, with subsequent abnormal trophoblast invasion and placentation, are probably the main etiologic factor of the increased incidence of pregnancy complications in PCOS (6). However, PCOS is also associated with an increased cardiometabolic risk (1), albeit clinical evidence about the related mortality is still lacking.

The current Research Topic includes 11 papers, selected after an intense reviewing process. Five articles represent original research, whereas the other 6 consisted of narrative and systematic reviews with or without meta-analysis.

In an experimental animal study, Feng et al. assessed the therapeutic effect and mechanism of action of *Dendrobium officinale* extract, which is commonly administered in China as a dietary supplement, in letrozole-induced PCOS rats. *Dendrobium officinale* extract reduced body weight, and restored estrous cycle by improving follicle development and lowering testosterone levels. These effects were potentially mediated by gut microbiota changes. Shen et al. assessed the effect of curcumin, a phenolic compound with potent anti-inflammatory and antioxidant properties also administered as a dietary supplement. The meta-analysis of 7 randomized controlled trials (RCTs) demonstrated that curcumin is safe and effective to reduce body weight, to improve inflammation, and markers of insulin resistance and lipid profile. Unfortunately, many trials had a high risk of bias, are from the Asiatic area and included a very low number of randomized subjects. Similarly, Rong et al. reported the synthesis of data from 16 RCTs for a total of 1385 patients showing the safety and efficacy of the *Guizhi fuling* pill as an adjuvant treatment for infertile patients with PCOS. When added to Western medicine, including oral contraceptives, insulin-sensitizing drugs, and clomiphene citrate, *Guizhi fuling* pill, a traditional Chinese herbal formula, induced a significant improvement in the ovulation and pregnancy rates of about 24% and 53%, respectively, in PCOS patients. Again the suboptimal certainty of evidence and the unclear risk of bias precluded any clear-cut recommendation for clinicians. A narrative review by Shawky discussed the available experimental and clinical data on the increased cardio-metabolic risk in offspring of women with PCOS exploring the different theories supporting that risk and analyzing the sex differences. Clearly, the increased cardiometabolic risk in offspring of mothers with PCOS probably needs the interaction of environmental (not only prenatal but also postnatal) and genetic factors over the lifespan. A sex interaction cannot be excluded as mediator of cardiometabolic outcomes. Gao et al. firstly performed a systematic review with meta-analysis, including 18 studies (case-control and cohort studies) for a total of 1265 PCOS patients and 894 controls. These studies aimed to evaluate the potential role of apelin and chemerin, two newly identified adipokines widely expressed in different organs. The data synthesis demonstrates that only chemerin, but not apelin, is significantly higher in PCOS patients suggesting that chemerin may be a therapeutic target to reduce the cardiovascular risk in patients with PCOS. Acupuncture is more and more used as a complementary and alternative treatment for infertility with controversial data regarding its efficacy (7). A comprehensive systematic review by Ye et al. on the use of acupuncture in women with PCOS underlined that all available meta-analyses agree that clinical evidence about the effectiveness of acupuncture for improving fertility is scarce in PCOS patients. Therefore, robust and well-designed clinical trials are needed to confirm or refute these hypotheses of efficacy. In the current Research Topic, the effect of acupuncture in PCOS patients under metformin administration was tested by Chen et al. in a systematic review with meta-analysis of 9 RCTs including a total of 1159 women. Even if acupuncture improved the ovulation and pregnancy rate by about 30% in PCOS patients with a potential action on the insulin resistance, the risk of bias was high for many studies and the quality of the evidence was defined as low to very low (8). Another interesting RCT by Zhang et al. assessed the effect of canagliflozin, a sodium-glucose cotransporter-2 (SGLT-2) inhibitor, a novel class of hypoglycemic oral drugs that enhance the renal glucose loss in 51 women with PCOS. Three months of canagliflozin administration induced only a significant, but clinically modest, beneficial effect on total testosterone and glucose concentrations.

Surprisingly, only one paper was published in this Research Topic about evidence-based treatment for infertile PCOS patients, such as letrozole administration (9, 10). The retrospective analysis by Shi et al. on a total of 539 infertile patients with PCOS, showed that letrozole administered starting on day 5 following menstrual bleeding is associated with a cumulative conception rate about 10% higher in comparison with administration on day 3 due to a potentially better endometrial thickness.

During the last years, the follicular output rate (FORT) has acquired a progressively more important role for scientists and clinicians as a marker of good reproductive prognosis. FORT is the ratio of pre-ovulatory follicle count on the trigger day to the antral follicule count (AFC), and is considered an important criterion by which to quantify the follicular development potential (11). Jiang et al. retrospectively analyzed the effects of FORT on reproductive performance in 454 infertile patients with PCOS scheduled for IVF cycles and showed that FORT is directly related to the cumulative pregnancy and live birth rates when the FORT was less than 70%. After stratification analyses, each additional unit of FORT increased the cumulative live birth rate by 1.3% confirming that FORT may be used as a simple and non-invasive tool to assess the dynamic changes of follicular growth in response to exogenous gonadotropins in the clinical practice.

Finally, in the current Research Topic, Bibi et al. published for the first time a link between some specific mitochondrial DNA mutations and PCOS using next-generation sequencing (NGS). Six regions with common variations in all analyzed genomes were identified, even if individual variations were also reported. Interestingly, the score-based pathogenicity analysis of mitochondrial variants that demonstrated frameshift mutations in the ND2 gene was associated to the highest risk for PCOS, potentially opening the door to predisposition tests for PCOS.

In conclusion, the reading of the current Research Topic, beside the scientific value of the specific papers published, arouse in us two main considerations. First, very few conclusions may be drawn from systematic reviews after considering the suboptimal quality of many of the studies available. Second, there is great interest from Asian scientists in regard to PCOS and its management in conjunction with alternative and adjuvant treatments. We hope that this Research Topic may stimulate the design of well-structured and powered RCTs, also in Western countries, to confirm or refute these new, interesting, and potentially useful strategies of treatment for women with PCOS.

## Author contributions

SP and DD conceptualized the study, acquired the main data, drafted the article, provided their final approval of the version to be published and agree to be accountable for all aspects of the work especially regarding its accuracy and integrity.
